# The Relationship between Serum Endocan Levels and Depression in Alzheimer's Disease

**DOI:** 10.1155/2016/8254675

**Published:** 2016-01-26

**Authors:** Kyung Hee Yoon, So Yeon Kim, Yoo Sun Moon, Daeyoung Roh, Sang Kyu Lee, Do Hoon Kim

**Affiliations:** ^1^Department of Psychiatry, Chuncheon Sacred Heart Hospital, Hallym University College of Medicine, Chuncheon 200-704, Republic of Korea; ^2^Institute for Skeletal Aging, Chuncheon Sacred Heart Hospital, Hallym University College of Medicine, Chuncheon 200-704, Republic of Korea

## Abstract

*Objectives*. Growing evidence suggests that angiogenic vascular factors may be involved in the pathogenic mechanism of Alzheimer's disease (AD), and recently endocan has been proposed as an angiogenic biomarker. The aim of this study was to measure serum endocan levels according to the presence of depression in AD and to investigate the association among the serum endocan levels, cognitive function, and depression in these patients.* Methods*. Serum endocan levels were measured in 26 AD patients with depression, 29 AD patients without depression, and 29 healthy controls using an enzyme-linked immunosorbent assay kit. The Mini-Mental State Examination-Korean version (MMSE-KC) and the Korean version of the Geriatric Depression Scale-Short Form (SGDS-K) were used to evaluate cognitive function and depressive symptoms, respectively.* Results*. Serum endocan levels were significantly lower in AD patients with depression than in AD patients without depression or healthy controls. Serum endocan levels were negatively correlated with SGDS-K scores but not with MMSE-KC scores in AD patients.* Conclusions*. This study suggests that serum endocan levels might be associated with depression in AD. Future studies are needed to investigate the pathophysiological mechanisms or the role of endocan in AD with depression.

## 1. Introduction

Although amyloid plaques, among other amyloid peptide fragments, have been identified as the primary pathological lesions in Alzheimer's disease (AD), it is not clear how these plaques are formed in the brain. It has been reported that angiogenic activation of the brain endothelium in AD leads to deposition of *β*-amyloid plaques and neurotoxic peptides are then secreted, which kill cortical neurons [1]. Growing evidence suggests that angiogenic vascular factors may be involved in the pathogenic mechanism of AD [1–3]. A number of studies have measured vascular endothelial growth factor (VEGF) levels in AD patients, but their results were inconsistent [3–5]. Recently, we found that serum VEGF levels were significantly increased in AD patients who had comorbid depression, which indicates that VEGF may be associated with depression in AD [6].

Interestingly, genetic expression of endocan is regulated by VEGF [7, 8], and endocan has also been proposed as a marker of angiogenesis [9, 10]. Endocan is a novel molecule which is specific to human endothelial cells. A number of studies have shown that endocan is overexpressed in multiple types of cancers [8, 11–17], which suggests that endocan could be involved in angiogenesis in various types of tumors and tumor growth. Moreover, increasing evidence has recently indicated that endocan may play a role in endothelium-dependent pathological disorders [18–23] and inflammatory diseases [24–28]. These findings suggest that endocan may be involved in endothelial dysfunction and inflammatory pathology.

We consider that endocan is possibly related to the pathophysiology of AD. However, no previous study has investigated the serum endocan levels in AD or any possible association between these levels and depression in AD. Therefore, we measured the serum endocan levels in AD patients with and without depression and attempted to verify the association among the serum endocan levels, cognitive function, and depression.

## 2. Methods

### 2.1. Subjects and Procedures

The subjects were recruited from the Dementia Clinic of Chuncheon Sacred Heart Hospital. The diagnosis of AD was based on the* Diagnostic and Statistical Manual of Mental Disorders*,* Fourth Edition* (DSM-IV) and neuropsychological tests that used the Korean version of the Consortium to Establish a Registry for Alzheimer's Disease Assessment Packet (CERAD-K). The diagnosis of depression in AD patients was based on the criteria proposed by Olin et al. [29] who developed a scale to identify the presence of depression in AD more precisely than the previously used DSM-IV criteria.

The subjects comprised 26 AD patients with depression (AD + depression), 29 AD patients without depression (AD − depression), and 29 healthy controls, all more than 65 years of age. Study protocols were reviewed and approved by the Ethics and Medical Research Committee of Chuncheon Sacred Heart Hospital, and all subjects gave written informed consent.

We excluded patients with inflammatory disease, malignancy, vascular dementia, other neurodegenerative diseases, diabetes mellitus, atherosclerosis, fracture, or history of substance abuse and patients who were taking medicines that contained acetylsalicylic acid or 5 alpha-reductase inhibitors, which are known to influence serum angiogenic factors. None of the subjects were taking any medicines except for antihypertensive agents. Current smoking status was established, and height and body weight were measured to calculate body mass index.

### 2.2. Measures

#### 2.2.1. Neuropsychological Assessment

The subjects were examined by a trained neuropsychologist. The Mini-Mental State Examination in the Korean version of CERAD Assessment Packet (MMSE-KC) [30] was used to assess cognitive function; the scale ranges from 0 to 30, and higher scores indicate better cognitive function. AD severity was measured with the Clinical Dementia Rating (CDR) [31], where CDR 0 = no dementia, CDR 0.5 = questionable dementia, CDR 1 = mild dementia, CDR 2 = moderate dementia, and CDR 3 = severe dementia, respectively. Depression severity was measured with the Korean version of the Geriatric Depression Scale-Short Form (SGDS-K) [32], which consists of 15 yes-or-no items about depressive symptoms; scores range from 0 to 15, with a higher score indicating more severe depression.

#### 2.2.2. Serum Endocan

Blood samples were drawn from an antecubital vein in a sitting position under nonfasting conditions. Samples were stored in plain tubes without ethylenediaminetetraacetic acid (EDTA) and were centrifuged at 1610 g for 10 min at 4°C. In order to reduce any technical or personal bias, the sampling was performed by two well-trained medical technologists in a standardized manner. The samples were collected during the study and stored at −80°C until use, for a maximum time period of 1 year. Serum endocan levels were measured with an enzyme-linked immunosorbent assay kit (ELISA; LUNGINNOV Systems, Lille, France). The assay range of the Endocan ELISA kit was 0.3–10 ng/mL. Intra-assay and interassay coefficients of variation for the endocan assay were <4.40% and <7.59%, respectively. The limit of quantification for the assay was 0.3 ng/mL.

### 2.3. Statistical Analysis

Demographics and clinical data were compared among the groups using one-way analysis of variance (ANOVA) and chi-square tests. Differences in serum endocan levels among the groups were measured using ANOVA, and Duncan's test was used for the post hoc analysis. Pearson's correlation analyses were performed between serum endocan levels and SGDS-K and MMSE-KC scores. Statistical analyses were performed using the Statistical Package for the Social Sciences (IBM, SPSS version 20.0). *p* < 0.05 was considered statistically significant.

## 3. Results

### 3.1. Demographic Characteristics and Clinical Data

The demographic characteristics and clinical data of the study population are presented in Table 1. There were no significant differences among the groups with respect to age, sex distribution, hypertension, body mass index, or smoking status. The mean MMSE-KC scores were significantly different; specifically, the mean score was lower in AD patients than in healthy controls (*F*(2,81) = 166.00, *p* = 0.000). The mean CDR score was significantly higher in AD patients than in healthy controls (*F*(2,81) = 61.32, *p* = 0.000). The mean SGDS-K score was significantly higher in the AD + depression group than in either the AD − depression group or healthy controls (*F*(2,78) = 111.71, *p* = 0.000).

### 3.2. Serum Endocan Levels

**Figure 1:**
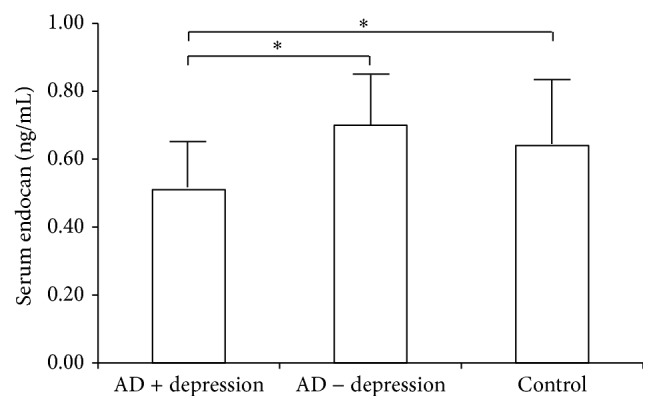
Serum endocan levels in AD + depression (*n* = 26), AD − depression (*n* = 29), and control (*n* = 29) groups. The AD + depression group had lower serum endocan levels compared with either of the two groups (*F*(2,81) = 7.57, *p* = 0.001). ^∗^ *p* < 0.05 on Duncan's post hoc test. The bars indicate standard deviations.

Serum endocan levels were significantly lower in the AD + depression group compared with the AD − depression group and healthy control group; levels in each group are shown in Figure 1. Endocan levels in the AD + depression, AD − depression, and healthy control groups were 0.51 ± 0.13, 0.70 ± 0.18, and 0.64 ± 0.20 ng/mL (mean ± SD), respectively. Post hoc comparisons of endocan levels indicated that these levels were lower in the AD + depression group than in the AD − depression group and the healthy controls (*F*(2,81) = 7.57, *p* = 0.001).

### 3.3. Correlations among Serum Endocan Levels, Depressive Symptoms, and Cognitive Function Scores

**Figure 2:**
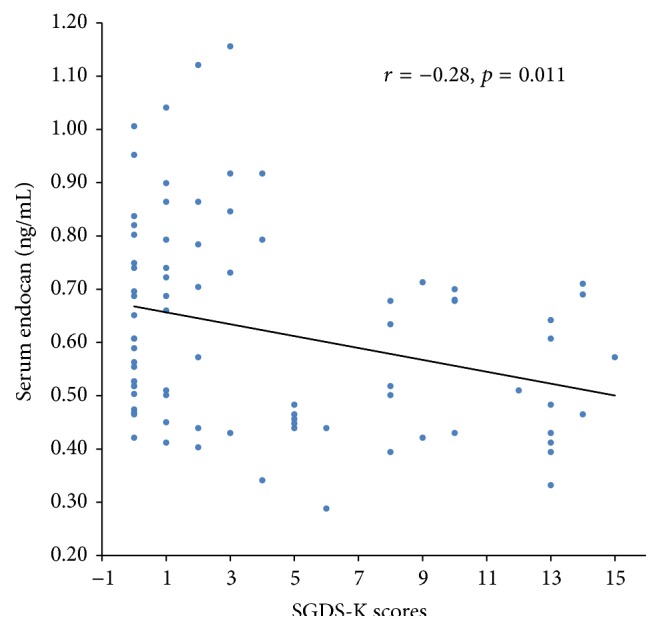
Scatter plot for the negative correlation between serum endocan levels and SGDS-K scores (*r* = −0.28, *p* = 0.011).* Note*. SGDS-K, Korean version of the Geriatric Depression Scale-Short Form.

Endocan levels were negatively correlated with SGDS-K scores in AD patients (*r* = −0.28, *p* = 0.011) (Figure 2). There was no significant correlation between endocan levels and the MMSE-KC scores (*r* = 0.02, *p* = 0.878).

## 4. Discussion

This study measured, for the first time, serum endocan levels in AD patients, and these levels were lower in the AD + depression group than in either the AD − depression group or the healthy control group. Our analysis showed a negative correlation between serum endocan levels and depressive symptoms in AD, suggesting that serum endocan may be closely related with depressive symptoms in AD.

The underlying mechanism in the relationship between serum endocan levels and depression in AD is unknown. As a probable mechanism, it can be suggested that increased cortisol levels in depression influence serum endocan levels. It is well known that late-life depression is associated with increased activity of the hypothalamic-pituitary-adrenal (HPA) axis [33], and hypercortisolemia was found in older depressed patients [34]. It was reported that cortisol inhibited endocan gene expression in preadipocytes and endocan production in adipocytes [35]. The results of this current study, based on the relationships outlined above, suggest that serum endocan levels might be decreased due to the inhibitory effect of elevated cortisol on endocan production in AD + depression group.

Another assumption is that the serum endocan levels might be regulated by inflammatory cytokines that are related to depression. Depressive patients have higher levels of a number of these cytokines, such as tumor necrosis factor-*α* (TNF-*α*) [36–38] and interferon-*γ* (IFN-*γ*) [39–42], and some studies have shown that cytokines such as IFN-*γ* and TNF-*α* influence endocan expression in endothelial cells. IFN-*γ* inhibited endocan mRNA, which was induced by TNF-*α* in human umbilical vein endothelial cells (HUVECs). When IFN-*γ* was combined with TNF-*α*, endocan mRNA was more strongly inhibited [43]. Another study reported that IFN-*γ* reduced endocan secretion, which was induced by TNF-*α* in HUVECs [44]. Therefore, in our study, decreased serum endocan levels in the AD + depression group could be related with the inhibitory effect of IFN-*γ* on endocan levels. Elevated level of IFN-*γ* in depression might reduce endocan secretion. However, we did not directly examine the relationship between endocan and inflammatory cytokines in patients with AD + depression. Besides, the correlation between endocan and inflammatory cytokines like C-reactive protein (CRP) has also been reported [19, 26, 35]. Additional studies should be conducted to determine this direct relationship and correlation between endocan and inflammatory cytokines in AD patients with depression.

The clinical significance of decreased serum endocan levels in patients with AD + depression is unclear. It has been reported that endocan may exert anti-inflammatory and vasculoprotective effects, which may play a role in the process of atherosclerosis [35]. Plasma endocan was decreased in obesity, and it was suggested that low plasma endocan levels may correspond to the loss of the vasoprotective factor in obesity [35]. Previous studies have shown that both vascular impairment [45, 46] and inflammation [47–50] can play a role in the progression of AD and that depression comorbidity in AD contributes to poor AD prognosis [51, 52]. Therefore, in this study, we reasoned that significantly decreased serum endocan levels in patients with AD + depression might have reduced the vasoprotective effects or anti-inflammatory activity, which could contribute to poor AD prognosis. Thus, this finding implicates that endocan might be a biomarker associated with pathophysiology in the AD + depression and a predictor of disease progression. The pathophysiological mechanisms and role of endocan in patients with AD + depression should be investigated.

Additionally, in our study, a significant negative correlation was observed between serum endocan levels and depressive symptoms in AD; hence, endocan could be a biomarker for depression severity in AD. It would be of interest to establish whether endocan is related with depressive symptoms in other depressive disorders such as major depression and dysthymic disorder, and thus additional studies on this relationship are also recommended.

This study has some limitations. Healthy control group has a relatively small sample size compared with that of AD group. Further studies with a larger sample may be needed for generalization of these results. Another limitation is that depressive patients without AD were not included in the study. To identify whether the presence of depression itself significantly influences the serum endocan levels, additional studies should be performed to examine the relationship between serum endocan and depressive symptoms in the elderly depressed patients. Finally, because this study had a cross-sectional design, we could not evaluate whether serum endocan levels would be altered with improvement of depression. To determine the possibility of endocan as a biomarker (trait or state marker) of depression in AD, studies assessing the serum endocan levels as the depression treatment progresses are needed.

## 5. Conclusions

This is the first study to suggest that serum endocan levels may be associated with depression in AD patients and that endocan might be a biomarker for the severity of depression in AD. This study will promote future studies investigating the pathophysiological mechanisms underlying the role of endocan in patients with AD + depression or other depressive disorders such as major depression and dysthymic disorder.

## Figures and Tables

**Table 1:** Demographic characteristics and clinical data of study subjects.

Variables	AD + depression (*n* = 26)	AD − depression (*n* = 29)	Control (*n* = 29)	Statistics	*p*
Sex (male/female)	4/22	7/22	11/18	*χ* ^2^ = 3.70	0.157
Age (mean ± SD)	76.88 ± 7.86	76.83 ± 5.66	73.83 ± 4.24	*F* = 2.39	0.098
Hypertension (*N*, %)	15 (57.69 %)	19 (65.52 %)	13 (44.82 %)	*χ* ^2^ = 2.69	0.260
BMI (mean ± SD)	22.53 ± 2.47	21.83 ± 2.60	23.35 ± 3.02	*F* = 2.29	0.108
Smoking status (*N*, %)	4 (15.38 %)	3 (10.34 %)	1 (3.45 %)	*χ* ^2^ = 2.30	0.316
SGDS-K (mean ± SD)	10.15 ± 3.50	2.65 ± 2.48	0.52 ± 0.83	*F* = 111.71	0.000
MMSE-KC (mean ± SD)	16.00 ± 3.46	14.90 ± 3.56	27.90 ± 1.59	*F* = 166.00	0.000
CDR (mean ± SD)	0.96 ± 0.37	1.10 ± 0.60	0.00 ± 0.00	*F* = 61.32	0.000

*Note.* AD + depression, Alzheimer's disease with depression; AD − depression, Alzheimer's disease without depression; Control, healthy control; SD, standard deviation; BMI, body mass index; SGDS-K, Korean version of the Geriatric Depression Scale-Short Form; MMSE-KC, Mini-Mental State Examination-Korean version; CDR, Clinical Dementia Rating.

## Abbreviations

AD + depression: Alzheimer's disease with depression
AD − depression: Alzheimer's disease without depression
Control: Healthy control
VEGF: Vascular endothelial growth factor
ANOVA: One-way analysis of variance
SD: Standard deviation
BMI: Body mass index
MMSE-KC: Mini-Mental State Examination-Korean version
SGDS-K: Korean version of the Geriatric Depression Scale-Short Form
CDR: Clinical Dementia Rating
TNF-*α*: Tumor necrosis factor-*α*
IFN-*γ*: Interferon-*γ*
HUVECs: Human umbilical vein endothelial cells.
